# Cytokines' Role in the Pathogenesis and Their Targeting for the Prevention of Frozen Shoulder: A Narrative Review

**DOI:** 10.7759/cureus.36070

**Published:** 2023-03-13

**Authors:** Ahmed Alghamdi, Ali H Alyami, Raad M. M Althaqafi, Ahmed Alzeyadi, Faisal S Alrubaei, Almuhanad A Alyami, Mohamed S Singer, Abdulelah A Saati, Wasn T Alotaibi, Maha O Alsharif

**Affiliations:** 1 Orthopedic Surgery, Al-Baha University, Al-Baha, SAU; 2 Surgery/Muscloskeletal Oncology, Limb Reconstructive Surgery, Sport Medicine and Arthroscopy, King Saud bin Abdulaziz University for Health Sciences, Jeddah, SAU; 3 Orthopedic Surgery, King Abdulaziz Specialist Hospital, Taif, SAU; 4 College of Medicine, Taif University, Taif, SAU; 5 Orthopedics, King Saud bin Abdulaziz University for Health Sciences, Ryad, SAU; 6 Orthopedics, Alhada Military Hospital, Taif, SAU; 7 Orthopedics, Taif university, Taif, SAU; 8 Orthopedics, King Faisal Medical Complex, Taif, SAU

**Keywords:** candidates, drugs, cytokines, shoulder, frozen

## Abstract

Frozen shoulder (FS) is a common name for shoulder movement limitation with different degrees of shoulder rigidity and pain. It is characterized by varying developmental courses, different levels of shoulder movement limitation, and background ambiguity due to the multiplicity of its causative factors. Systemic inflammatory cytokines monitoring and restraining is easy to apply, fast to conduct, and needs lower costs compared to invasive methods for frozen shoulder stage evaluation and early controlling of its progress to the stage that necessitates surgical intervention. The aim of this review was to assess the recent findings concerning the role of cytokines in FS pathogenesis and the possibility of preventing or controlling their progress through targeting these cytokines by the new drugs candidates, such as hyaluronan (HA), botulinum toxin type A (BoNT A), Tetrandrine, tumor necrosis factor-stimulated gene-6 (TSG-6), and cannabidiol. Searching the PubMed site, we encountered out of 1608 records, from which 16 original studies were included for the quantitative construction of this systematic review screening of the recent studies to investigate the different FS pathogenic pathways. Most of the scenarios are centered around the inflammatory and fibrotic process triggered by synovial and capsular fibroblast stimulation. This mechanism depends mainly on alarmins cytokines, including thymic stromal lymphopoietin (TSLP), interleukin-33 (IL-33), and interleukin-25 (IL-25), with the stimulation of interleukin-1 α (IL-1α), interleukin-1 β (IL-1β), tumor necrosis alpha (TNF-α), cyclooxygenase-1 (COX-1) and cyclooxygenase-2 (COX-2) in a joint capsule. Different pathways of transforming growth factor- β (TGF-β) stimulation, resulting in overexpression of the fibrotic factors as tenascin C (TNC), fibronectin 1, collagen I (COL 1) and collagen III (COL III), and matrix metalloproteinases (MMPs) in the capsular or synovial/capsular fibroblasts. The overall investigation of these studies led us to conclude that the new drug candidates proved their efficiency in controlling the common pathogenesis of the inflammatory and fibrotic pathways of frozen shoulder and therefore represent a prospect for easy and early controlling and efficiently treating this serious disease.

## Introduction and background

Frozen shoulder (FS) is a common name for the shoulder movement range limitation associated with different degrees of shoulder rigidity and pain. It is characterized by varying developmental courses, different levels of shoulder movement limitation, and background ambiguity due to the multiplicity of its causative factors. Worldwide, frozen shoulder prevalence is estimated to be about 2-5% [1]. The most vulnerable individuals to frozen shoulder affection are diabetics, as 30% of the patients are expected to experience FS once or more in their lifetime especially over the age of 50, in addition to hypothyroidism which causes a 21% prevalence, especially in women [2]. Other conditions, such as Dupuytren contracture, autoimmune diseases, and trauma beside breast cancer treatment, have been linked to the occurrence of frozen shoulder [3]. Idiopathic frozen shoulder is currently conceived as a suddenly developed restriction of shoulder range of motion with an unknown cause, not linked to a defined condition, and showing unremarkable radiographic changes [4]. A new body of evidence has recently emerged, leading to a change in the concept of the idiopathic frozen shoulder being not linked to any disease to its probable occurrence due to local joint or systemic disturbance. In this context, the inflammatory markers, such as tumor necrosis factor alpha (TNF-α), cyclooxygenase-1 (COX-1), cyclooxygenase-2 (COX-2), interleukin-1α (IL-1α), interleukin-1β (IL-1β), protein expression were found to be considerably elevated in the idiopathic frozen shoulder joint capsules [5]. 

Moreover, the most frequent secondary frozen shoulder cases are those associated with diabetes mellitus, hypothyroidism, Dupuytren contracture, and breast cancer treatment [6, 3], which are commonly associated with immune system perturbation and increased inflammatory status. In spite of the previous belief of being a self-limiting disorder, frozen shoulder is recently considered to be an essential need for medical intervention in a high percentage of patients [7]. However, the decision of whether the frozen shoulder case is to be considered for medical intervention or to be left for self-resolving depends on the pain degree, the disease stage, the shoulder movement disability level, and the triggers of the pathophysiology. Whatever the reason for the frozen shoulder development, it follows a common pathophysiology course characterized by a local or systemic cytokines up-regulation. The levels of the shoulder structure deterioration and the time passed from the start of the complaint to the time of the patient's presentation determine the method of the intervention. Considering cytokines are the core of the frozen shoulder pathophysiology, the early prevention of their stimulus or controlling their induction can limit its progress to the stage that necessitates surgical intervention. Compared to invasive methods, systemic inflammatory cytokines monitoring is cheaper, fast, and easy to apply for evaluating frozen shoulder stage and for deciding the proper clinical intervention. This narrative review aims to summarize the findings from recent studies concerning the role of cytokines in the pathogenesis and the possibility of preventing or controlling FS progress through targeting of these cytokines by some new drug candidates, such as hyaluronan (HA), botulinum toxin type A (BoNT A), Tetrandrine, tumor necrosis factor-stimulated gene-6 (TSG-6), and cannabidiol.

## Review

Material and methods

Multiple searches were made to collect the published articles of relevant concern using the following words as key search inputs in MEDLINE and PubMed databases in the last 15 years. The research articles and literature reviews in only the English language were included after they were evaluated and met the needed criteria for this review. The search inputs keywords, the resulting number of publications, and the links for each search input are listed in Table 1. Focus was made on the articles published in the last 15 years concerning the relation between frozen shoulder and cytokines or factors that play roles in the case pathogenesis or candidates drugs that target some pathway of the disease progress. Articles were accepted to be included if there was a complete discussion on the link between FS pathophysiology and any type of cytokines or factors that take part in the progress. Experiments on animal models that meet the aim of the study were included, while reviews, case reports, and imaging studies were excluded.

**Table 1:** The different search entries, their outcome abstract numbers, and their search links IL - interleukin; CRP - C-reactive protein; TNF - tumor necrosis factor

Search inputs	Results No.	Link
Frozen shoulder, inflammation	553	https://pubmed.ncbi.nlm.nih.gov/?term=frozen+shoulder%2C+inflammation
Frozen shoulder, cytokines	89	https://pubmed.ncbi.nlm.nih.gov/?term=frozen+shoulder%2C+cytokines
Frozen shoulder, IL	103	https://pubmed.ncbi.nlm.nih.gov/?term=frozen+shoulder%2C+IL
Frozen shoulder, TNF	23	https://pubmed.ncbi.nlm.nih.gov/?term=frozen+shoulder%2CTNF
Frozen shoulder, diabetes	287	https://pubmed.ncbi.nlm.nih.gov/?term=frozen+shoulder%2C+diabetes
Frozen shoulder, obesity	423	https://pubmed.ncbi.nlm.nih.gov/?term=frozen+shoulder%2C+obesity
Adhesive Capsulitis, inflammation	543	https://pubmed.ncbi.nlm.nih.gov/?term=+++Adhesive+Capsulitis%2C+inflammation
Adhesive Capsulitis, CRP	30	https://pubmed.ncbi.nlm.nih.gov/?term=Adhesive%20Capsulitis%2C%20CRP&page=3
Frozen shoulder, cortisone	53	https://pubmed.ncbi.nlm.nih.gov/?term=frozen+shoulder%2Ccortisone
Frozen shoulder, corticosteroid intra-articular injection	232	https://pubmed.ncbi.nlm.nih.gov/?term=frozen+shoulder%2C+corticosteroid+intra-articular+injection
Frozen shoulder, sodium hyaluronate injection	35	https://pubmed.ncbi.nlm.nih.gov/?term=%E2%80%9Cfrozen+shoulder%2C+sodium+hyaluronate+injection
Total	2122	

Results

**Figure 1:**
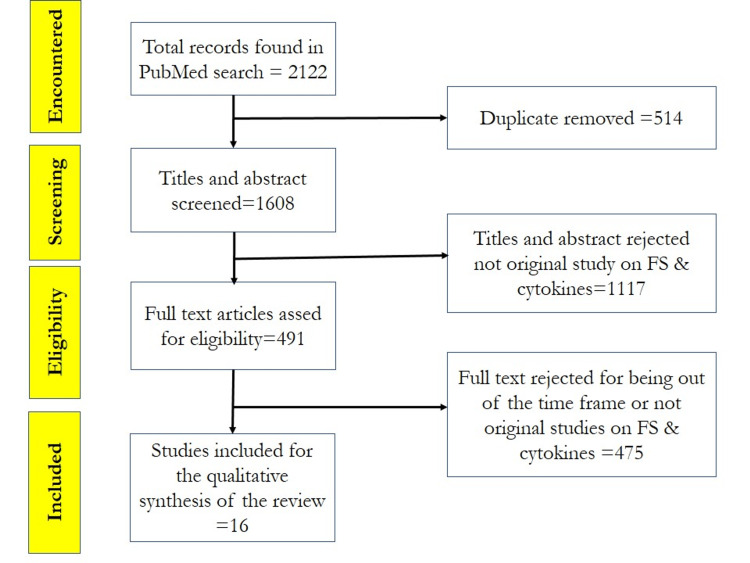
PRISMA flow chart used for review construction The process of filtering and choosing the title included in the construction of the review according to Preferred Reporting Items for Systematic Reviews and Meta-Analyses (PRISMA) FS - frozen shoulder

A total number of 2122 titles were calculated from all search entries. After the removal of duplicates, a total of 1608 studies were available for screening. All possible search entries keywords, the search links for each entry, and the resulting number of articles for each entry are listed in Table 1. Verification of the full text article containing data on the FS pathogenesis and cytokines resulted in 16 eligible articles that were accepted for the qualitative construction of this review. A chart of the applied process for the selection of the eligible studies according to Preferred Reporting Items for Systematic Reviews and Meta-Analyses (PRISMA) is presented in Figure 1. The number of frozen shoulder clinical cases, number of controls, biopsy location, and the methods used for disease marker analysis, the most prominent results and the conclusions for each study are listed in Table 2.

**Table 2:** Data included Authors and year, the studies designs, the number of patients/ or experimental animals and the control, the measured markers, the methods of measurement, the results and the concluded remarks for each study. NF-κB - ;IL - interleukin; MMP3 - metalloproteinase-3; VEGF - vascular endothelial growth factor; IHC - immunohistochemistry; qPCR - quantitative polymerase chain reaction; FS - frozen shoulder; TGF-β - transforming growth factor beta; PDGF-A - platelet-derived growth factor subunit A; RT-PCR - reverse transcription polymerase chain reaction; TNF-α - tumor necrosis factor alpha; COX - cyclooxygenase; ELISA - enzyme-linked immunosorbent assay; BoNT-A - botulinum toxin type A; TA - triamcinolone acetate; ROM - range of motion; TNC - tenascin C; qRT PCR - real-time quantitative reverse transcription quantitative polymerase chain reaction; FN1 - fibronectin 1; TGFB - transforming growth factor beta; TGFBR - transforming growth factor beta receptor 1; AGEs - advanced glycation end products; VCAM - vascular cell adhesion protein; PDPN - podoplanin; CCL-20 - chemokine ligand 20; CRP - C-reactive protein; TSH - thyroid-stimulating hormone; RAGE - receptor for advanced glycation end-products; LCG - liquid charomatography; HMGB1 - high mobility group protein B1; CCL - coracohumeral ligament; TLR - Toll-like receptor; CHL - coracohumeral ligament; IGHL - inferior glenohumeral ligament, α‐SMA - α‐smooth muscle actin; PPAR‐γ - peroxisome proliferator- activated receptor gamma; CTR - calcitonin receptor; PKC - protein kinase C; PKA - protein kinase A; BAX - Bcl-2-associated X protein; IFN-γ - interferon- gamma; TRAF - transient receptor potential ankyrin; sCT -salmon calcitonin; SCF - synovial/capsular fribroblast

Author and year	Study design and evidence levels	No. of		Measured markers	Methods of analysis	Results	Conclusions
		Patients	Controls				
Kanbe et al., 2009 [8]	Case series + in vitro cell culture; level III	10	-	NF-κB, IL-6, MMP3, β1-integrin and VEGF in synovial tissues between the long head of the biceps tendon	IHC	Localization of NF-κB, CD29 (β1-integrin) in vascular tissue, and IL-6 around vascular tissue, MMP-3 and VEGF on the surface layer of synovial tissue	Association of NF-κB, IL-6, MMP3, β1-integrin and VEGF expression in the synovial tissue with frozen shoulders development
Kabbabe et al., 2010 [9]	Case-control; level IV	13	10	Cytokines IL-6, IL-8, and MMP3 in synovial tissue	qPCR	High cytokines mRNA expression in FS than control	Severity of FS is correlated to cytokines expression
Nago et al., 2010 [10]	Case series + in vitro cell culture; level III	7	-	FS-synovial fibroblast proliferation and Procollagen α1, TGF-β, PDGF-A expression	RT-PCR and histology	FS-synovial fibroblast proliferation and expression of these adhesion-related cytokines were inhibited by hyaluronan treatment	Hyaluronan inhibit fibroblast proliferation and cytokines expression in FS patients
Lho et al., 2013 [11]	Case-control; level III	14	7	IL-1α, IL-1β, TNF-α, COX-1 and COX-2 expression in joint capsule and subacromial bursa	RT-PCR, ELISA, and IHC	FS patients have higher expression of IL-1α, IL-1β, TNF-α, COX-1 and COX-2 in joint capsule and IL-1α, TNF-α and COX-2 in the subacromial bursa, compared to control	Higher expression of these cytokines in joint capsule and subacromial bursa is associated with FS development
Joo et al., 2013 [12]	Case-control; level III	15	13	Pain intensity - numeric rating scale (NRS) and range of motion (ROM)	Manual goniometer	Botulinum toxin type A (BoNT-A) has comparable results similar to the steroid triamcinolone acetate (TA) in improving the shoulder ROM and pain score	Intra-articular BoNT-A can be used as an alternative for TA to avoid the steroid-induced side effects or as a second-line agent, for patients who have failed to respond to steroids
Cohen et al, 2016 [13]	Case-control; level III	9	8	mRNA expression of tenascin C (TNC), fibronectin 1 (FN1), and TGFB receptor in the FS synovial/capsular fibroblast	qRT-PCR	FS patients have higher expression of TNC, FN1, and TGFBR in the synovial/capsular fibroblast compared to the control	TNC, FN1, and TGFb1 receptor may play roles in FS pathogenesis and their high expression in the synovial/capsular fibroblast can be taken as markers of FS development.
Hwang et al., 2016 [14]	Case-control; level II	8	14	AGEs in the FS capsule	IHC	FS patients have higher expression of AGEs in the FS capsule compared to the control	A potential role for AGEs in FS pathogenesis and explain the fibroblastic proliferation and deposition of collagen matrix in idiopathic frozen shoulder
Cher et. al., 2018 [15]	Case-control; level IV	10	10	Alarmin shoulder capsule tissue samples	IHC	FS patients have higher alarmin expression in the joint capsule compared to the control	FS pain severity is associated with the expression of the danger molecules as alarmin
Akbar et al., 2019 [16]	Case-control; level IV	10	10	Fibroblast activation markers CD248, CD146, VCAM and PDPN and cytokines IL-6, IL-8, and CCL-20 in shoulder capsule samples	qPCR, IHC, and ELISA	Fibroblast activation markers and cytokines IL-6, IL-8 & CCL-20 were highly expressed in the RI rotator interval of FS patients compared to the control	Fibroblasts activation and cytokines dysregulation are key players in the fibrotic and inflammatory process of FS disease
Park et al., 2020 [17]	Case-control; level III	202	606	Serum CRP, fasting glucose level, HbA1c, dyslipidemia, TSH, and free T3	-	FS patients have higher CRP serum levels compared to control	High Serum CRP >1.0 mg/L is a marker for FS supporting the FS link to chronic systemic inflammation
Yano et al., 2020 [18]	Case-control; level III	33	25	RAGE, HMGB1, TLR2, TLR4 & NF-κB gene expression coracohumeral ligament (CHL) and anterior inferior glenohumeral ligament (IGHL)	RT-PCR, IHC, and LCG	FS patients have high expression of RAGE, HMGB1, TLR2, TLR4, and NF-kB in the CHLs and IGHLs compared to the rotator cuff tear group	FS pathogenesis may occur through AGEs and HMGB1 binding to RAGE and activating NF-kB signaling pathways. Therefore targeting of this pathway could be beneficial for FS treatment
Chen et al., 2021 [19]	Laboratory study (rat); level III	6	6	The expression of COX 1, COL3, COL3, IL‐1β, and TNF‐α, vimentin, and α‐Smooth muscle actin (α‐SMA) In shoulder joint fluid and joint capsule	ELISA, IHC, rPCR, and western blotting	The PPAR‐γ agonist rosiglitazone ameliorated the FS pathogenesis through the downregulation of COX 1, IL‐1β, COL1, COL3, TNF‐α, vimentin, and α‐SMA	Downregulation of inflammatory and fibrotic cytokines by PPAR‐γ agonist can be a promising way for FS control and treatment
Yang et al., 2020 [20]	Case-control and cell culture; level III	9	10	Calcitonin receptor, collagen type I (COL1A1), COL3A1, fibronectin 1, laminin 1, (TGF‐β1), and (IL‐1α), VEGF and IL‐6 in synovial/capsular fibroblasts exposed to different conc. of salmon calcitonin in synovial and capsular tissues	RT-PCR, IHC, flow cytometry	FS-SCFs, treatment with increasing conc. of sCT causes downregulation of the mRNA expression of adhesion molecules as COL1A1, COL3A1, fibronectin 1, laminin 1, TGF‐β1, and IL‐1α, and stimulated the expression of the VEGF and IL‐6	Salmon calcitonin decreased the adhesion ability of FS-synovial/capsular fibroblast by interacting with its surface- CTR through the downstream PKC or PKA pathway
Liu et al., 2021 [21]	Case-control and cell culture; level III	5	-	TNF-α-stimulated gene/protein 6 (TSG-6) ability of capsule fibroblasts regulation in frozen shoulder pathogenesis Through the expression of Bcl-2, COL1A1, TNF-α, IL-6, IL-1β, TGF-β1, BAX and phosphorylated Smad2 in shoulder capsular bursa tissue	RT-PCR, immunoconfocal, western blotting, ELISA, and flow cytometry	TSG-6 treatment decreased fibroblast viability, accelerated its apoptosis, and decreased the expression of Bcl-2, COL1A1, TNF-α, IL-6, IL-1β, TGF-β1 and phosphorylated Smad2, and increased BAX	TSG-6 protein downregulated FS capsular fibroblast activation through inhibition of the TGF-β/Smad2 signaling pathway
Akbar et al., 2021 [22]	Case-control; level III	10	10	Expression of IL-17, MMP1, MMP3, Bcl2, IL-6, IL-8, Bax, IFN-γ, and cell viability in frozen shoulder tissue	Flow cytometry, qRT-PCR, western blotting, and ELISA	Immune cell population switches from a predominantly macrophage in normal tissue to a T cell-rich environment in FS and increases IL-17-producing T cells that are able to induce inflammatory and fibrotic state in FS through elevating IL- 17R through s TRAF-6/NF-κB dependent	Inhibition of IL-17 or its pathway can be therapeutic target for controlling FS pathogenesis
Zhao et al., 2021 [23]	Controlled laboratory study (rat); level III	16	16	Protein Expression of TGF-β1, Collagen 1, Collagen II and Collagen 1V, COX-2, PGE-2 IL-1β, IL-6, TNF-α, and MMP-3 rat's rotator cuff muscles	IHC, ELISA, and western blotting	Tetrandrine inhibited the FS-associated increased protein expression of TGF-β1, collagen 1, collagen II, collagen 1V, COX-2, PGE-2 IL-1β, IL-6, TNF-α, MMP-3 and TIMP-1	Tetrandrine administration is effective for prevention of FS pathogenesis due to its anti-inflammatory, anti-angiogenic, and antifibrotic effects

Shoulder Joint Motion Range Limitations

Glenohumeral capsule contraction was accused of being the cause of the FS-associated restriction of the joint passive range of motion (ROM) [24]. The normal passive ROM of the shoulder joint which is estimated to be 15-20 ml, was reported to be decreased in cases of FS to only 5º [25, 26]. The use of the botulinum toxin type A (BoNT-A), a new drug candidate, has improved the ROM from 6º in FS patients to 22º in treated patients [12]. The FS cases start with synovial hypervascularisation and hypertrophy, leading to shoulder pain that is present at rest and magnified by movement [27,28]. This early synovial inflammation changing to a fibrotic state leads to progressive ROM restriction in all direction especially internal rotation, abduction, and external rotation [29]. The following stage of the FS is characterized by pain intensity reduction with fibrosis and thickening of joint capsule leading to joint capsular volume reduction [30] and rotator interval structures contracture associated with multidirectional restriction of glenohumeral ROM [31].

Frozen Shoulder Pathophysiology Course

Whether as idiopathic or secondary to other diseases, frozen shoulder development starts with the early overexpression of inflammatory cytokines [11, 32]. High mobility group box 1 (HMGB1) proteins, a member of the damage-associated molecular pattern (DAMP) or alarmins, are released into the extracellular matrix (ECM) when cells are distressed or destroyed [33]. This triggers the inflammatory response leading to the induction of the proinflammatory cytokines as interleukin-1 (IL-1), interleukin-6 (IL-6), tumor necrosis factor alpha (TNF-α), transforming growth factor-β (TGF-β1), and platelet-derived growth factor (PDGF) in the joint fluid [11, 32, 33]. The FS severity was associated with the levels of cytokines expression [15]. High levels of these cytokines are known to stimulate fibroblast proliferation and differentiation and hence disturbance of collagen synthesis [14]. Frozen shoulder development was correlated to the expression of cytokines, such as nuclear factor kappa B (NF-κB), IL-6, matrix metalloproteinase-III (MMP3), β1-integrin, and vascular endothelial growth factor (VEGF) in the synovial tissue [8]. Compared to the control, there was a higher expression of IL-1α, IL-1β, TNF-α, COX-1, and COX-2 in the joint capsule and IL-1α, TNF-α, and COX-2 in the subacromial bursa [11]. Moreover, frozen shoulder severity was directly proportional to the levels of cytokines expression in the synovial tissue [9].

Impact of New Drug Candidates on Frozen Shoulder

Restraining of cytokines induction in the joint environment is to be targeted for efficient controlling of the FS development and progression. Among the frozen shoulder conservative treatment, oral and intra-articular corticosteroid injection was implemented with considerable efficiency [34].

However, intra-articular injection of steroids was accused of being associated with delayed effects on the healing of a rotator cuff [35]. To avoid these undesirable effects, an alternative drug, botulinum toxin type A (BoNT-A) intra-articular injection, was shown to improve FS pain score and ROM with keeping the desirable effects of corticosteroids [12]. Another drug, hyaluronan, was recently shown to inhibit synovial adhesion-related cytokines expressions as procollagen α1, transforming growth factor - β (TGF-β), and platelet-derived growth factor A (PDGF-A), and hence suppress fibroblast proliferation; therefore, hyaluronan was considered as a candidate drug for FS restraining [10]. Vimentin, TNF‐α, IL‐1β collagen 1 (COL1), and collagen 3 (COL3) are key effectors of FS pathogenesis and development. The peroxisome proliferator-activated receptor gamma (PPAR‐γ) agonist rosiglitazone was recently demonstrated to alleviate FS pathogenesis through these cytokines down regulation of [19]. Salmon calcitonin (sCT), a new candidate drug through its high ability to stimulate calcitonin receptor, activates protein kinase C (PKC) and protein kinase A (PKA) that are responsible for the down-regulation of fibrosis‐related molecules such as collagen 1, collagen 3, TGF‐β1, and interleukin-1α [20]. The fibrotic pathways in the course of frozen shoulder development were also demonstrated to be inhibited by another candidate drug, Tetrandrine, which was shown to down-regulate the protein expression of TGF-β1, collagen 1, collagen II, collagen IV, COX-2, prostaglandin E2 (PGE-2), IL-1β, IL-6, TNF-α, MMP-3, and the tissue inhibitors of metalloproteinases (TIMP-1) [23]. 

Discussion

The primary or secondary frozen shoulder initiation and progress have been known to include various factors that contribute mainly to the general body inflammatory and fibrotic process, in addition to some factors that are specific to the joint environment. The deciphering of these main factors sharing in the FS pathogenesis and the recent trial for their therapeutic targeting may help determine the proper way to control the disease development and its progress. This systematic review investigated 16 studies on 1122 individuals with frozen shoulder or synovia/capsular fibroblast from cases with FS. Collectively the so far known factors that contribute to FS pathogenesis are various and huge and mainly revolve around inflammatory and fibrotic pathways. In the course of generalized or local inflammation, cytokines are a cornerstone in FS development and, therefore, they can be taken as markers for disease development. Of these factors, IL-1α, IL-1β, TNF-α, COX-1, and COX-2 in the joint capsule and IL-1α, TNF-α, and COX-2 in the subacromial bursa were reported to be overexpressed [11]. Due to its ability to induce immune cell proliferation, recruitment, and blood vessel vasoconstriction [36], IL-1 is well known to have a central role in acute and chronic inflammation. In the joint capsule or the glenohumeral joint synovium, IL-1𝛼, IL-1𝛽, IL-6, and TNF-𝛼 elevation play important parts in FS pathogenesis [11, 9, 37 ]. When human TNF-𝛼 was transgenically overexpressed, severe rheumatic arthritis spontaneously developed concomitantly with systemic inflammatory conditions characterizing TNF-𝛼 as a common key player of inflammation [38]. Moreover, IL-1β, IL-6, and TNF-α induction were associated with FS development [23, 21]. IL-8 was found to be overexpressed in fibroblast cultured from FS capsule [16] and synovial tissue of FS patients in the context of the local inflammatory process, while interleukin-17 (IL-17) was reported to induce inflammatory and fibrotic state and was found to be induced in FS [39]. Transforming growth factor-β (TGF-β) is a multifunctional cytokine-induced ECM protein and was shown to contribute in the inducement of fibrosis [40]. This main fibrotic inducer was found to be elevated in the anterosuperior capsule of FS compared to nonspecific synovitis and was suggested to act as a constant stimulus for the induction of fibrosis in the joint capsule [33]. More importantly, the serum level of TGF-β was elevated in the serum of FS patients compared to control [41] and thus is suggested to be taken as a marker for FS development. The fibrotic effect of TGF-β was identified to be induced through the TGF-β/Smad pathway [42] due to the association of its high levels with tissue fibrosis [43]. Transforming growth factor β (TGF-β1) elevation is linked to the severity of renal fibrosis that occurs in the course of injuries caused by diabetes, hypertension, aging, and ischemia [44]. The urinary TGF-β1 levels are positively correlated with renal fibrosis as it reflects the degree of renal pathology and CKD progression [45]. Fibroblast activation can be enhanced by the platelet-derived growth factor (PDGF) in the context of FS development [33], while TGF-β- Smad2/3 signaling in cardiac fibroblasts was further demonstrated to be a main pathway of the fibrotic response [46]. 

Alarmins are a group of cytokines that were first characterized to be released by bronchial epithelium cells when being insulted by dangers from the environment, such as allergens, pollutants, bacteria, and viruses. These alarmins cytokines, including thymic stromal lymphopoietin (TSLP), interleukin-33 (IL-33), and interleukin-25 (IL-25) [47,48], are released to alarm the innate and adaptive immune system to react to these insulting dangers by some mechanism that elect the type 2 asthma through dendritic cells activation to direct T helper 2 (Th2) cells expansion and stimulation of group 2 innate lymphoid cells proliferation (ILC2) [39]. Of these danger molecules, alarmin was shown to be highly expressed in FS joint capsules [15]. The secretion of these cytokines occurs when airway epithelial cells undergo injuries caused by several environmental triggers, such as allergens, respiratory viruses, bacteria, cigarette smoking, and airborne pollutants [49]. Cyclooxygenase enzymes have main roles in inflammation through their ability to convert arachodenic acid to prostaglandins that are responsible for the pain sensation. Furthermore, frozen shoulder was reported to express high levels of COX1 and COX2 in the capsule and COX2 in the subacromil bursa [32]. Multiple factors concerting in the FS fibrosis process as tenascin C (TNC), fibronectin 1 (FN1), collagen (COL1), and collagen 3 (COL3), were found to be overexpressed in the FS capsule or synovial/capsular fibroblasts [10, 13]. Moreover, the expression of collagens types, MMPs, and TGFs were induced in FS pathogens [23, 22]. In a considerable percentage of cases, FS pathogenesis follows the systemic chronic inflammatory process; hence, it was linked to elevated serum C-reactive protein (CRP) and hemoglobin A1c (HbA1c) [17]. Among the chronic inflammatory process inducers, advanced glycation end products (AGEs) have been included in the factors that result in FS pathogenesis as AGEs expression have been reported to be elevated in the capsule of FS [15]. Therefore the incidence of FS in diabetic persons has been recorded to be much higher than its incidence in the general population [50]. This was clearly proved by the elevation of the expression of the AGEs receptor (RAGE), the high mobility group box 1 (HMGB1), the Toll-like receptor (TLR2), TLR4, nuclear factor-kappa B (NF-κB) in the FS coracohumeral ligament (CHL) and anterior inferior glenohumeral ligament (IGHL) [18]. 

Targeting these inflammatory and fibrotic factor pathways may enable to focus on the proper remedy for prevention, control, or treatment of FS. This study stipulates the different candidates for FS treatment, which are the following.

Hyaluronan

Hyaluronan (HA), a polysaccharide found in joint synovial fluid, was proved to be effective in fibrotic joint treatment either through intra-articular injection [51]. This antifibrotic effect of hyaluronan has been recently found to be through inhibition of the fibrotic pathway, including suppression of the mRNA expression of TGF-B, PDGF in the fibroblast of the joint glenohumoral synovial and capsule and therefore decreasing cell proliferation and expression of adhesion-related procollagen [10]. This hyaluronan-related study has led to the conclusion that it is a candidate for preventing FS development and progression.

Botulinum Toxin Type A

Botulinum toxin type A (BoNT A) is a neurotoxic protein synthesized and released by *Clostridium botulinum* bacteria that acts by preventing the neurotransmitter acetylcholine exocytosis from the nerve endings at the synapse with muscles' cells or with nociceptors neurons resulting in muscle flaccidity and reduction in pain sensation, in addition to its ability to reduce neurogenic inflammatory process [52]. More importantly, BoNT A has a fibrosis-inhibiting ability through suppression of TGF-B and arresting the fibroblast cell cycle [53]. It has been found to improve shoulder ROM and a pain score of 17. Therefore, BoNT A is considered a valid candidate for FS alternative therapy. 

Salmon Calcitonin

Salmon calcitonin (sCT) nasal spray was reported to produce rapid FS pain relief and noticeable shoulder ROM [54, 55]. This sCT-induced FS improvement was recently found to be through the sCT-CTR interaction on Synovial/Capsular fibroblast surface associated with the reduction of the mRNA expression of fibrosis‐related molecules collagen type I, Type III A1, fibronectin 1, laminin 1, TGF‐β1, IL‐1α in sCT-dose dependent manner and upregulation of Vascular Endothelial Growth Factor (VEGF) and IL‐6 mRNA expression [20]. sCT was shown to induce its anti-inflammatory effect comparable to hyaluronan through downregulation of the mRNA expression of nuclear receptor 4A1 (NR4A1), nuclear receptor 4A2 (NR4A2), nuclear receptor 4A3 (NR4A3), and MMPs in human cell lines [56]. Therefore, sCT can be considered an alternative candidate for FS treatment.

Tetrandrine

Tetrandrine has been shown to have a higher antifibrotic effect than prednisolone through suppression of the fibrotic factors collagen type I-III, TGF-B, and Smad 3 mRNA in rabbits' ear scar tissue [57]. Recently, Tetrandrine was proven to have anti-inflammatory, antifibrotic, and anti-angiogenesis effects in the rate capsular tissue through which it prevented the FS progress, and therefore it was suggested to be a secure and efficient remedy for FS progress restraining and its cure [22].

Tumor Necrosis Factor-Stimulated Gene-6

Tumor necrosis factor-stimulated gene-6 (TSG-6) was shown to suppress fibrosis in keloid tissue by reducing keloid fibroblast proliferation and enhancing its apoptosis through TGF-B1 /Smad signaling pathway interference [58]. This anti-fibrosis effect was more clearly shown to suppress capsular fibroblast proliferation and accelerate its apoptosis through decreasing the expression of Bcl-2, COL1A1, TNF-α, IL-6, IL-1β, TGF-β1, and phosphorylated Smad2, and increasing BAX expression [21]. Therefore, TSG-6 was suggested to be a potential candidate alternative as a remedy to restrain FS development.

Tezepelumab

An anti-alarmin monoclonal antibody tezepelumab competes with TSLP and prevents it from binding to its receptor [59], through which it downregulates respiratory allergies. In the context of the FS treatment clinical trials, tezepelumab has not yet been examined and there is still a need for evaluation as a possible candidate for an alternative drug for FS control and treatment.

Cannabidiol

Cannabidiol is one of the cannabis sativa extract constituents that have neuroprotective and anti-inflammatory effects without the undesired psychotropic effects of cannabis sativa extracts due to its low binding affinity to cannabinoid receptors [60]. It was demonstrated to have anti-inflammatory and pain relieving effects [61]. Cannabidiol was recently shown to decrease IL-6, IL-8, and MMP3 production by rheumatic arthritis synovial fibroblast through binding to transient receptor potential ankyrin 1 (TRPA1), and by raising intracellular calcium concentration and opening of mitochondrial permeability transition pore leading to reduction of fibroblast viability, therefore it was suggested to have an anti-arthritic effect [62]. This newly emerged drug can be a potential candidate for developmental control and amelioration of frozen shoulder.

## Conclusions

Frozen shoulder pathogenic pathways are mainly through inflammatory and fibrotic processes by synovial and capsular fibroblast stimulation depending on alarmin cytokines, including thymic stromal lymphopoietin (TSLP), interleukin-33 (IL-33), and interleukin-25 (IL-25), with the stimulation of IL-1α, IL-1β, TNF-α, COX-1 and COX-2 in joint capsules. These different pathways of cytokines induction lead to TGF-β stimulation that ends with the overexpression of the fibrotic factors, such as tenascin C (TNC), fibronectin 1, collagen I, collagen III, and MMPs in the capsular or synovial/capsular fibroblasts. The new drug candidates proved their efficiency in controlling these inflammatory and fibrotic pathways and represent a hope for easy and early restraining and effective treatment for this serious disease.
